# The Iranian version of the Copenhagen Psychosocial Questionnaire (COPSOQ) for assessment of psychological risk factors at work

**DOI:** 10.15171/hpp.2017.03

**Published:** 2016-12-18

**Authors:** Mohammad Aminian, Iman Dianat, Anvar Miri, Mohammad Asghari-Jafarabadi

**Affiliations:** ^1^Department of Occupational Health, Tabriz University of Medical Sciences, Tabriz, Iran; ^2^Road Traffic Injury Research Center, Tabriz University of Medical Sciences, Tabriz, Iran; ^3^Department of Psychology, Farhangian University, Kurdistan Pardis Shahid Modares, Sannadaj, Iran; ^4^Tabriz Health Services Management Research Center, Tabriz University of Medical Sciences, Tabriz, Iran; ^5^Department of Statistics and Epidemiology, Faculty of Health, Tabriz University of Medical Sciences, Tabriz, Iran

**Keywords:** Psychometric, Feasibility, Reliability, Validity, Iran

## Abstract

**Background: ** The Copenhagen Psychosocial Questionnaire (COPSOQ) is a widely used tool for evaluation of psychosocial risk factors at work. The aims of this study were to describe the short version of Farsi COPSOQ and to present its psychometric properties.

**Methods:** A total of 427 administrative health care staff participated in this descriptive methodological study. Forward–backward procedure was adopted to translate the questionnaire from English into Farsi. Content validity was assessed by a panel of 10 experts. Construct validity was evaluated by exploratory and confirmatory factor analyses. The internal consistency and test-retest reliability were assessed using Cronbach’s α and intraclass correlation coefficient(ICC), respectively. The feasibility was assessed using ceiling and floor effect.

**Results:** The short version of Farsi COPSOQ was configured with 16 dimensions (32 items).Content validity of the questionnaire was established. Factor analysis supported the conceptual multi-dimensionality (four factors), and therefore confirmed the construct validity of the Farsi COPSOQ. The internal consistency (Cronbach’s α ranging between 0.75 and 0.89) and test retest reliability (ICC values ranged from 0.75 to 0.89) were both approved and the results showed no ceiling or floor effect.

**Conclusion:** The results support the use of Farsi COPSOQ for evaluation of psychological risks and for research purposes in Iranian population.

## Introduction

The psychosocial work environment is generally regarded as one of the most important aspects of the work environment in present and future societies.^1^ Evidence suggest that a high percentage of employees in both developed and developing countries are exposed to psychosocial stressors at their work environment, with the consequences of exposure to such stressors are believed to be very substantial for employees, work places, and society.^1-4^ Some of these consequences are cardiovascular diseases, musculoskeletal symptoms, mental problems, depression, stress, increased sickness absence and labor turnover, and decreased reduced quality of life, motivation and work productivity.^5-8^, Therefore, a better understanding on the work-related psychosocial factors may have a significant impact on safety and health promotion at work and on productivity enhancement, as well.

Work-related psychosocial risks are related to the design and management of work systems and their social and organizational contexts, which have a great potential to cause psychological or physical harm.^9^ The psychosocial risks concern to a large number of variables regarding the interaction between the individual, collective, and organizational dimensions of professional activities.^10^ Thus, the implications go beyond the individual workers/employees and concern the working groups and the organization as well. This problem seems to be worsening with increasing intensity and density of working tasks, the use of new communication methods, and the increasing working demands in nowadays working systems. Therefore, it is important to understand the individuals at their working environment in terms of their interactions with other people, employees and organizational contents that make up that environment.

There are several theoretical approaches that have been developed for evaluation of psychological risks.^1,11,12^ However, most of these measures are not comprehensive (e.g. dealing with one specific theory) and do not cover different components of the psychosocial work environment. Among these, the Copenhagen Psychosocial Questionnaire (COPSOQ) developed by the Danish National Institute of Occupational Health has desirable features that make it an appropriate tool in this regard.^1^ The COPSOQ is a tool that includes most of the dimensions of the influential psychosocial theories and is not just based on one theory. The COPSOQ was developed around the year 2000 (in Danish) and has been available in English since 2005. The cross-cultural adaptation of the English language questionnaires seems to be necessary for use in other societies. The linguistic validation and psychometric properties of the different versions of the COPSOQ in other countries with different languages are well documented.^10,13,14^ Therefore, it was decided in this study to validate the Farsi version of the COPSOQ for people with Persian (Farsi) language. The objective of this study was to develop and evaluate the psychometric properties of a Farsi version of the COPSOQ.

## Materials and Methods

### Study design, setting, and participants

All administrative health care workers (in hospitals and health centres) in the city of Bukan–Iran, in 2015, were invited to participate in this descriptive-methodological study. Being a full-time office worker with at least one-year job experience and having no chronic mental or physical problem (by self-report) were considered as inclusion criteria for this study. A total of 427 administrative health care staff declared their agreement to participate in this study. Some investigators have suggested five or more participants per item or a total sample size of 200 participants as appropriate for factor analysis,^15-17^ and therefore this represented a good sample size in this study. Data were collected using the short version of the COPSOQ. Demographic details of the study participants including their age, gender, and educational level were also recorded. The study period was between June and August 2015.

### Copenhagen Psychosocial Questionnaire 

The first version of the COPSOQ was developed in 1997 by the Danish National Research Centre for the Working Environment as a standardized questionnaire to cover a broad range of psychosocial factors. There were three versions of the COPSOQ questionnaire including long research version (with 141 questions and 30 dimensions), medium-length version (for work environment professionals with 95 items and 26 dimensions) and short version (for workplaces with 44 items and 8 dimensions).^1^ Although the first version of the COPSOQ covered the main dimensions of the different theories in occupational health psychology, it failed to address some work-related aspects such as justice, trust and rewards. The second version of the COPSOQ was, therefore, a response to this limitation.^18^ The short version of the COPSOQ was used in this study. The respondents to the COPSOQ are asked to respond to the questions using items on a 5-point scale, most of which are as A=Always, B = Often, C=Sometimes, D=Seldom, and E = Never/Hardly or as A=To a very large extent, B=To a large extent, C=Somewhat, D=To a small extent, and E=To a very small extent. A simple scoring system is applied in this scale so that the scores for each of the items (which are 0, 1, 2, 3 or 4) in each scale are simply added.

The Farsi conversion of the COPSOQ was performed using a forward–backward translation process. The forward translation was conducted by two specialists in psychology. Then, the back translation to the original English of the Farsi version was carried out by two Persian professional translators. The English back–translation was then compared with the original edition and minor revisions were made on the Farsi version. For potential linguistic problems, the questionnaire was completed and evaluated by 50 subjects and minor revisions were made following their feedback.

For qualitative evaluation, the questionnaire was reviewed for content validity by an expert panel of 10 psychologists, ergonomists and occupational health specialists and few items were modified following their feedback. For quantitative evaluation, a survey containing questions (based on a 4-point scale) in two general sections was delivered to the expert panel members. The first section included questions about relevancy, clarity and simplicity of the items, and was used to for calculation of content validity index (CVI). The second section had a question with regard to the necessity of each item, and was used to compute content validity ratio (CVR). CVI and CVR values higher than 0.75 and 0.62, respectively, were considered to be suitable with respect to the number of expert panel members.^19^

### Statistical analysis

Data were presented as mean ± standard deviation (SD) for the COPSOQ scale and quantitative variables and *n* (%) for qualitative variables. Intraclass correlation coefficient (ICC) and Cronbach’s α were calculated for assessing stability reliability and internal consistency of the scale, respectively, and values >0.7 was considered as appropriate.^20^ Percentage of scores at the boundaries of the scaling was used for assessing ceiling and floor effects.^21^

Structure detection, which is to examine the underlying (or latent) relations between variables, was assessed using exploratory factor analysis (EFA). EFA was performed by principal axis factoring (PAF) extraction method and using direct oblimin rotation with Kaiser normalization. The study applied the scree plot procedure to determine the number of factors to be extracted.^20^ For the evaluation of model sufficiency, Bartlett’s test of sphericity, Kaiser-Meyer-Olkin (KMO) measure of sampling adequacy and total variance explained were used. KMO values > 0.7 generally show the suitability of the factor analysis for the data. Bartlett’s test of sphericity is to test the hypothesis that a correlation matrix is an identity matrix, which shows that variables are unrelated and consequently not appropriate for structure detection. Small values (<0.05) of the significance probability show a satisfactory factor analysis. Factor loading values ≥0.3 were considered as significant relationship between items and factors.^22^ Confirmatory factor analysis (CFA) was used to evaluate the fit between EFA extracted model and observed data. Asymptomatic covariance matrix was a weighted matrix, while input matrix was covariance matrix of data. Fit indices and rational values of the indices were as: χ^2^/df <5, root mean square error of approximation (RMSEA) <0.08, goodness of fit index (GFI), comparative fit index (CFI), adjusted goodness of fit index (AGFI) > 0.9. Statistical analysis was carried out using SPSS 21.0 (SPSS Inc., Chicago, IL) and LISREL 8.80 (Scientific Software International Inc., 2007). *P* values < 0.05 were considered as statistically significant.

## Results

### Sample characteristics

There was a total of 450 administrative health care staff, from which 427 staff (249 males, 58.3%; 178 females, 41.7%) returned the completed questionnaires (response rate=94.8%). The age of participants ranged from 20 years to 60 years (mean=45.3 years; SD=5.2 years). The majority of participants were married (n=363, 85.0%) and had bachelor-level degree (n=195, 45.7%).

### Content validity

The COPSOQ was evaluated for content validity by a panel of 10 professional experts in both qualitative and quantitative manners. In the qualitative evaluation, experts provided written feedback on the clarity and relevancy of the content of the COPSOQ items to the Iranian culture. Some of the items were improved according to the qualitative suggestions of the expert panel and the content validity of the scale was generally supported in this stage. It is noteworthy that in the first step evaluation phase, 6 out of 44 items were revised following the quantitative results and qualitative recommendations. One item regarding the sexual harassment was also excluded from the questionnaire since most of the respondents did not provide answer to this question due to cultural backgrounds. For the quantitative evaluation of the content validity, CVI (ranged between 0.75 and 1.00) and CVR (ranged between 0.75 and 1.00) showed satisfactory results for each item and consequently for the COPSOQ (Table 1).

**Table 1:** The scores of CVI and CVR of the short version of Farsi COPSOQ

**Item**	**Item content**	**CVI**	**CVR**
5	Does your work put you in emotionally disturbing situations?	0.77	0.75
7	Do you have a large degree of influence concerning your work?	1.00	0.77
8	Can you influence the amount of work assigned to you?	0.75	0.92
11	Is your work meaningful?	0.83	0.95
12	Do you feel that the work you do is important?	0.80	0.83
13	Do you feel that your place of work is of great importance to you?	0.75	0.75
15	At your place of work, are you informed well in advance concerning for example important decisions, changes, or plans for the future?	0.83	0.77
16	Do you receive all the information you need in order to do your work well?	0.80	0.92
17	Is your work recognized and appreciated by the management?	0.75	0.77
18	Are you treated fairly at your workplace?	1.00	0.90
19	Does your work have clear objectives?	0.75	0.92
20	Do you know exactly what is expected of you at work?	0.95	0.77
21	To what extent would you say that your immediate superior gives high priority to job satisfaction?	0.90	0.90
22	To what extent would you say that your immediate superior is good at work planning?	1.00	0.95
23	How often is your nearest superior willing to listen to your problems at work?	0.82	0.83
24	How often do you get help and support from your nearest superior?	1.00	0.75
26	Do you feel that your work drains so much of your energy that it has a negative effect on your private life?	0.90	1.00
27	Do you feel that your work takes so much of your time that it has a negative effect on your private life?	0.85	0.83
28	Can you trust the information that comes from the management?	0.92	0.77
29	Does the management trust the employees to do their work well?	0.77	0.75
30	Are conflicts resolved in a fair way?	0.90	0.85
31	Is the work distributed fairly?	0.95	0.92
32	In general, would you say your health is?	0.83	0.77
33	How often have you felt worn out?	0.75	0.90
34	How often have you been emotionally exhausted?	0.95	0.95
35	How often have you been stressed?	0.90	0.83
36	How often have you been irritable?	1.00	0.75
39	If yes, from whom?	0.83	0.92
40	Have you been exposed to physical violence at your workplace during the last 12 months?	0.75	0.77
41	If yes, from whom?	0.85	0.90
42	Have you been exposed to physical violence at your workplace during the last 12 months?	0.92	0.95
43	If yes, from whom?	0.90	0.90

### Construct validity

EFA

In this analysis, KMO measures of sampling accuracy were 0.837, which justified the sufficiency of the model. The results of Bartlett’s test of sphericity (χ^2^ (496)=5640.9; *P*<0.001), which was used to justify the suitability of data for factor analysis, was in line with KMOs.^23^ The factor analysis gave four factors as follows:

- Factor 1: quality of leadership (items 21 and 22), social support from supervisors (items 23 and 24), rewards (items 17 and 18), justice and respect (items 30 and 31), trust (items 28 and 29), and predictability (items 15 and 16)
- Factor 2: self-rated health (item 32), burnout (items 33 and 34), stress (items 35 and 36), work-family conflict (items 26 and 27) and emotional demands (item 5)
- Factor 3: meaning of work (items 11 and 12), commitment to the workplace (item 13), influence at work (items 7 and 8) and role clarity (items 19 and 20)
- Factor 4: offensive behaviour (items 39 through 43).

The total variance explained was 47.51% (with 21.8%, 11.0%, 8.1% and 6.6% for factor 1 factor 2, factor 3 and factor 4, respectively). The items with low communalities (less than 0.2) were deleted from the analysis (items 1, 2, 3, 4, 6, 9, 10, 14, 25, 37, 38 and 44). The results of the analysis were, therefore, revised after deleting these items. Cut-off values higher than 0.3 for factor loadings suggest that all items have been strongly loaded on the Farsi COPSOQ (as shown in Table 2). It should also be noted that the above mentioned deleted items had small values in loadings. Additionally, the factors were correlated as a justification of using the Direct Oblimin Rotation method (corr > 0.3 was seen among factors).

**Table 2:** Factors and factor loading for each test item

Items	Item description	Factor 1	Factor 2	Factor 3	Factor 4
s23	Supervisor listens to problems	0.761			
s24	Supervisor talks about performance	0.749			
s21	Supervisor gives priority to job satisfaction	0.724			
s18	Treated workplace	0.719			
s22	Work planning	0.707			
s17	Recognized by management	0.686			
s30	Conflicts resolved fairly	0.649			
s29	Management trust employees	0.628			
s31	Work distributed fairly	0.607			
s28	Employees trust information	0.599			
s16	Information to work well	0.483			
s15	Informed about changes	0.427			
s34	Emotionally exhausted		0.835		
s33	Worn out		0.769		
s35	Stressed		0.721		
s27	Time conflict		0.575		
s36	Irritable		0.537		
s26	Energy conflict		0.536		
s32	Health discrimination		-0.504		
s5	Emotional disturbing		0.384		
s12	Work important			0.726	
s13	Workplace great importance			0.703	
s11	Work meaningful			0.585	
s19	Clear objectives			0.520	
s8	Amount of work			0.492	
s7	Influence work			0.481	
s20	Expectation			0.374	
s39	Threats of violence (1)				-0.662
s40	Threats of violence (2)				0.619
s41	Physical violence (1)				-0.601
s42	Physical violence (2)				0.581
s43	Bullying				-0.569

Extraction method: principal axis factoring with simple structure. Rotation method: Oblimin with Kaiser normalization.

### Ceiling and floor effects

Based on the results, there was no ceiling effect for none of the factors. However, a floor effect was observed for factor 4 (0.2%), which was less than 15%.

### CFA

**Figure 1:**
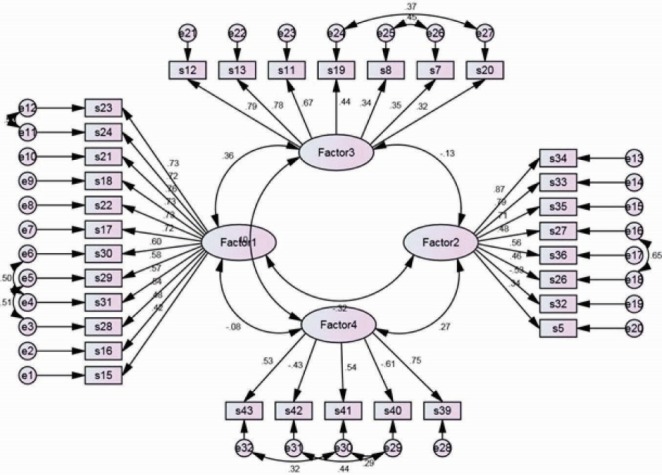
Relations between items and factors and between factors based on CFA. All relations between factors and items as well as between the factors were statistically significant (All *P* < 0.05). Factors names: quality of leadership, social support from supervisors, rewards, justice and respect, trust, and predictability (Factor 1), self-rated health, burnout, stress, work-family conflict and emotional demands (Factor 2), meaning of work, commitment to the workplace, influence at work and role clarity (Factor 3), and offensive behaviour (Factor 4). Fit indices: χ^2^/df=1.73 < 5, RMSR=0.062, RMSEA (90% CI) = 0.056 (0.040; 0.071), CFI=0.93, GFI=0.92 and AGFI=0.89.

The results of the CFA analysis indicated that the model fit was established reasonably by the indices (χ^2^/df=2.03 < 5; SRMR=0.061 < 0.1, RMSEA = 0.049 < 0.08 and 90% CI: 0.045 to 0.054, CFI=0.91 > 0.90, NFI=0.91 > 0.90, NNFI=0.91 > 0.91, GFI=0.90 > 0.91, AGFI=0.91 > 0.90), after some modifications in the model based on modification indices.^20,24,25^ Moreover, the relationships between parameters and factors were also evaluated based on this model. The factor loading values indicated that the items had significant loadings on the four factor solution, with the standardized factor loadings ranging from 0.32 to 0.84. This indicated that all items represented moderate to strong factor loadings^26^ (Figure 1).

Additionally, correlation between factor 1 and factor 2 (*P*<0.001), factor 1 and factor 3 (*P*<0.001), factor 2 and factor 3 (*P*=0.021), factor 2 and factor 4 (*P*<0.001) were all statistically significant. The results of EFA and CFA confirmed the models, and therefore the findings supported the construct validity of measure.

### Reliability

Internal consistency of the COPSOQ (evaluated by Cronbach’s α coefficient) and its test–retest reliability (evaluated by ICC) were found to be satisfactory. The internal consistency reliability of all factors was found to be good based on the results of Cronbach’s α (0.89, 0.82, 0.76 and 0.78 for factor 1, factor 2, factor 3 and factor 4, respectively). ICC for the scale (value = 0.95; 95% CI = 0.90 to 0.98) also showed a satisfactory (> 0.7) test-retest reliability. Reliability coefficients were also calculated for the four factors. The coefficients for the four factors were as follows: factor 1 (α = 0.89); factor 2 (α = 0.75); factor 3 (α = 0.76), and factor 4 (α = 0.78).

## Discussion

This study aimed to develop and validate of the Farsi version of the COPSOQ for the evaluation of psychosocial risk factors of the working environments in Iran and other Farsi-speaking communities. Studies on psychosocial problems and their consequences, which are not only important for individual employees/workers but also for different occupations and organizations, have not received adequate attention in Iran. This is because there are relatively limited reliable and valid tools and instruments in this country. This highlights the fact that Farsi-speaking communities are in great need for reliable and valid instruments to evaluate psychosocial factors, and consequently improve the design and management of working systems and their social/organizational contexts. The findings of the present study indicated that the Farsi version of the COPSOQ is a reliable and valid instrument for evaluating psychosocial factors, and that the psychometric properties of this tool are generally in agreement with the original English^1^ and other versions.^10,14^

Studies conducted on the validation of the COPSOQ in other countries generally involve long or medium-length versions of the questionnaire. The authors of the present study were interested in the short version of this questionnaire, partly because Iranian companies and organizations have not the same cultural background as other countries with respect to the questionnaire approach and workers and employees do not respond to many items. The authors also believed that this study is the first step which could consequently lead to the use of the long or medium-length version of this questionnaire by Iranians.

In reliability analyses, both internal consistency and test–retest reliability of the Farsi COPSOQ were shown to be good. The Farsi version of the COPSOQ indicated good internal consistency. The coefficients for the four factors ranged between 0.75 and 0.89. The Cronbach’s α coefficients found in this present study are relatively consistent with those reported for the original instrument (Cronbach’s α ranging from 0.61 to 0.81)^1^ and much better than that reported for the French version of the COPSOQ (Cronbach’s α ranging between 0.37 and 0.78).^10^ Additionally, the ICC value (ranging between 0.75 and 0.89) which assessed test-retest reliability of the Farsi COPSOQ, showed very good reproducibility of this measure. This high ICC value for the Farsi version of the COPSOQ highlights a strong stability of this measure over time. The original study has not reported the ICC for the COPSOQ. However, comparison of our results with the French version of the COPSOQ^10^ indicates that the ICC value in the present study is much higher than that reported for that study (ICC values between 0.37 and 0.78 for different subscales).

The results of the study indicated no ceiling effect or floor effect for the Farsi version of the COPSOQ. Floor or ceiling effects are matter of concern when more than 15% of the study participants achieve the lowest or highest possible score, respectively, which was not the case in our study. Thus, this finding confirms the feasibility of this measure in Iranian population. This is in agreement with the findings reported for the Spanish version of the COPSOQ.^14^

The content validity of the Farsi COPSOQ was approved by both qualitative (e.g. feedback from the expert panel members) and quantitative assessments (e.g. the agreement level between expert panel members, with CVR and CVI values greater than 0.75 and 0.70, respectively). Neither the original instrument,^1^ nor other study has not reported the use of CVR measure for essentiality of the items and CVI measure for the simplicity, relativity, and clarity of the subscales, and therefore it was not possible to compare the results.

With regard to the construct validity, the results of factor analysis, which was carried out to determine the number of factors that can be addressed by the Farsi COPSOQ, recognized four factors for this tool. The results of this study are in line with the findings reported by of the original study and also by others who have shown the multi-dimensionality of the COPSOQ.^1,10^

## Conclusion

This study was aimed to validate and culturally adapt the short version of the COPSOQ for the Iranian-language population. The results indicated high degrees of reliability, feasibility and validity for the Farsi COPSOQ as a tool for surveys of the psychosocial work environment to be used by the Iranian companies and workplaces. The psychometric properties of the Farsi version of the COPSOQ are consistent with those of the original English version, suggesting that this tool can be used by Iranian researchers for evaluation of psychological risks and for research purposes.

## Ethical approval

Consent to use the original COPSOQ was obtained by the authors. The ethics committee of the Tabriz University of Medical Sciences reviewed and approved the study protocol (Ethics code: TBZMED.REC.1394.910). All the study participants were informed about the aim of the study and were assured of the privacy of the records. All the participants gave their written consent before participation.

## Competing interests

The authors declare that there is no conflict of interests.

## Disclaimer

The authors claim that no part of this manuscript has been copied from other sources.

## Authors’ contributions

MA contributed to the design of the work and data acquisition. ID contributed to the conception and design of the work; interpretation of data for the work; and drafting the work. AM contributed to the analysis and interpretation of data for the work. MAJ contributed to the analysis and interpretation of data for the work.

## Acknowledgments

The authors would like to acknowledge all participants who collaborated in this study.
